# Dr. Textor

**Published:** 1861-05

**Authors:** 


GERMANY lately lost another of its distinguished men in the death of Dr. TEXTOR, formerly Professor of Surgery in the University of Würzburg. At the time of his death he was seventy-eight years of age. He was the author of an excellent work on surgery, and enjoyed an extensive reputation as an operator; being particularly known by his resections of the joints.

